# Diabetes alters vascular mechanotransduction: pressure-induced regulation of mitogen activated protein kinases in the rat inferior vena cava

**DOI:** 10.1186/1475-2840-5-18

**Published:** 2006-09-08

**Authors:** Kevin M Rice, Devashish H Desai, Sunil K Kakarla, Anjaiah Katta, Deborah L Preston, Paulette Wehner, Eric R Blough

**Affiliations:** 1 Department of Pharmacology, Physiology and Toxicology, Joan C. Edwards School of Medicine, Marshall University, Huntington, WV, USA; 2 Department of Cardiology, Joan C. Edwards School of Medicine, Marshall University, Huntington, WV, USA; 3 Department of Biological Sciences, Marshall University, Huntington, WV, USA

## Abstract

**Background:**

Diabetes mellitus is an important risk factor for increased vein graft failure after bypass surgery. However, the cellular and molecular mechanism(s) underlying vessel attrition in this population remain largely unexplored. Recent reports have suggested that the pathological remodeling of vein grafts may be mediated by mechanically-induced activation of the mitogen activated protein kinase (MAPK) signaling pathways and the MAPK-related induction of caspase-3 activity. On the basis of these findings, we hypothesized that diabetes may be associated with alterations in how veins "sense" and "respond" to altered mechanical loading.

**Methods:**

Inferior venae cavae (IVC) from the non-diabetic lean (LNZ) and the diabetic obese (OSXZ) Zucker rats were isolated and incubated *ex vivo *under basal or pressurized conditions (120 mmHg). Protein expression, basal activation and the ability of increased pressure to activate MAPK pathways and apoptosis-related signaling was evaluated by immunoblot analysis.

**Results:**

Immunoblot analyses revealed differential expression and activation of extracellular signal-regulated kinase (ERK1/2), p38 and c-Jun NH2-terminal kinase (JNK) MAPKs in the IVCs of diabetic rats as compared to non-diabetic rats. In particular, the expression and basal phosphorylation of p38β- (52.3 ± 11.8%; 45.8 ± 18.2%), JNK 1- (21.5 ± 9.3%; 19.4 ± 11.6%) and JNK3-MAPK (16.8 ± 3.3%; 29.5 ± 17.6%) were significantly higher (P < 0.05) in the diabetic vena cava. An acute increase in IVC intraluminal pressure failed to increase the phosphorylation of ERK1-, JNK-2, or any of the p38-MAPKs in the diabetic obese Zucker rats. Also, IVC loading in the LNZ led to a 276.0 ± 36.0% and 85.8 ± 25.1% (P < 0.05) increase in the cleavage of caspase-3 and caspase-9, respectively, with no effect on these molecules in the OSXZ. No differences were found in the regulation of Bax and Bcl-2 between groups. However, basal expression levels of Akt, phospho-Akt, PTEN, phospho-PTEN and phospho-Bad were higher in the diabetic venae cavae (P < 0.05).

**Conclusion:**

These data suggest that diabetes is associated with significant alteration in the ability of the vena cava to activate MAPK- and apoptosis-related signaling. Whether these changes are associated with the increased vein graft attrition seen in the diabetic population will require further investigation.

## Background

Diabetic patients form a major subgroup of patients undergoing cardiovascular interventional procedures. Compared to non-diabetics, diabetics show a higher incidence of vein graft neointimal hyperplasia along with significantly higher graft failure rates [1]. The reasons underlying this phenomenon are not entirely clear; however recent data suggest that diabetes influences endothelial and smooth muscle cell (SMC) signaling and protein expression [2]. The possible influence of such cellular changes on vein graft failure is not well understood.

In addition to factors related to diabetes, mechanical stimuli may also play a role in causing graft failure. Upon grafting, the vein segment is subjected to arterial blood pressure and the cells residing in the vascular wall are subjected to an increased stretch stimulus. Recent studies have shown that the average circumferential tensile stress in the graft wall can be increased by 140 times or higher compared with that in a native vein [3-5]. Chronic elevations in the mechanical forces experienced by vessel walls are thought to trigger adaptive processes leading to hyperplasia, hypertrophy, and inflammation [6]. In experimental vein grafts, mechanical stretch due to arterial pressures is associated with rapid disruption and degradation of α-actin filaments in SMCs along with SMC death within the first day after vein grafting surgery [3,4]. Although not fully understood, it is thought that this response is mediated, at least in part, by the activation of the p38 mitogen activated protein kinase (p38-MAPK) and the downstream apoptotic regulator caspase-3 [7].

The purpose of this study was to investigate whether inferior venae cavae obtained from normal and diabetic rats exhibit similar load-induced MAPK and apoptosis-related signaling. We hypothesized that biochemical mechanotransduction is altered in diabetic veins, which if true, may help elucidate the mechanism(s) by which diabetes adversely influences vein graft failure. To test this hypothesis, we examined the basal levels and arterial pressure-induced phosphorylation (activation) of the extracellular regulated protein kinase (ERK1/2)-, p38- and stress activated protein kinase (SAPK/JNK) MAPKs along with the regulation of the apoptotic mediators caspase-9, caspase-3, Bad, Bax and Bcl-2 in protein extracted from normal and diabetic venae cavae. The results suggest the diabetes is associated with alterations in the ability of the vena cava to activate MAPK- and apoptosis-related proteins. Taken together, these data may help explain why diabetes is associated with an increased risk of vein graft failure.

## Materials and methods

### Animals

All procedures were performed in accordance with the Guide for the Care and Use of Laboratory Animals as approved by the Council of the American Physiological Society and the Animal Use Review Board of Marshall University. All procedures were conducted in strict accordance with the Public Health Service policy on animal welfare. Young (10 week, n = 12) male normal lean Zucker (non-diabetic) and young (10 week, n = 12) male obese syndrome-X Zucker (diabetic) rats were obtained from the Charles River Laboratories and barrier housed one per cage in an AAALAC approved vivarium. Housing conditions consisted of a 12 H: 12 H dark-light cycle and temperature was maintained at 22 ± 2°C. Animals were provided food and water *ad libitum*. Rats were allowed to recover from shipment for at least two weeks before experimentation during which time the animals were carefully observed and weighed weekly.

### Materials

Antibodies against p-p44/p42 MAPK (Thr202/Tyr204) (mitogen activated protein kinase ERK 1/2) [cat #9106], p-p38 MAPK (Thr180/Tyr182) [cat #9216], p-SAPK/JNK (Thr183/Tyr185) [cat #9251], p44/p42 MAPK (mitogen activated protein kinase ERK 1/2) [cat #9102], p38 MAPK α [cat #9218], SAPK/JNK [cat #9252], Akt [cat #9272], p-Akt (Ser 308) [cat #9275 S], PTEN (phosphatase and tensin homolog) [cat #9552], p-PTEN (Ser 308/Thr382-383) [cat #9554S], caspase-3 [cat #9662], caspase-9 [cat #9506], p-BAD (Ser 136) [cat #9295], mouse IgG, and rabbit IgG antibodies were purchased from Cell Signaling Technology (Beverly, MA). Antibody against p38β (N-14) [sc-15918], Bax [sc-493], and Bcl-2 [sc-7382] antibodies and anti-goat secondary antibodies were from Santa Cruz Biotechnology (Santa Cruz, CA) were purchased from Santa Cruz. Antibody against p38γ [AF1347] was purchased from R&D Systems (Minneapolis, MN). Precast 10% and 15% SDS-PAGE gels were procured from Cambrex Biosciences (Baltimore, MD). Enhanced chemiluminescence (ECL) western blotting detection reagent was from Amersham Biosciences (Piscataway, NJ). Restore western blot stripping buffer was obtained from Pierce (Rockford, IL) and 3T3 cell lysates were from Santa Cruz Biotechnology (Santa Cruz, CA). All other chemicals were purchased from Sigma (St. Louis, MO).

### Inferior vena cava preparation

Rats were anesthetized with a ketamine-xylazine (4:1) cocktail (50 mg/kg ip) and supplemented as necessary for reflexive response. In a sterile, aseptic environment, the ventral surface of the thorax was shaved and the superficial musculature was exposed by means of a transverse incision through the skin distal to the thoracic cavity. After midline laparotomy and perforation of the heart, the inferior vena cava was isolated and the *in situ *length measured as the distance from the right atrium to the diaphragm. Inferior venae cavae were removed, including a section of the right atrium and the diaphragm, in which the latter were used for mounting the vessels. After removing the extra connective tissue venae cavae were cannulated onto polystyrene tubing (outside diameter 3.0 mm; inner diameter 2.6 mm) with the aid of a dissection microscope. Mounted vessels were secured using 4.0 silk sutures along the tissue segments from the atrium and the diaphragm. After mounting, the vessel length was adjusted with the aid of a micromanipulator to coincide with the *in situ *resting length. All dissection and mounting procedures were performed rapidly and with care to prevent stretching or tearing of the inferior venae cavae. Vessel manipulation was completed in oxygenated Krebs-Ringer bicarbonate (KRB) buffer maintained at 37°C. After mounting, venae cavae were allowed to equilibrate in the vessel chamber for at least one hour before pressure loading. To examine the effect of increased loading on inferior vena cava signal transduction, mounted vessels were subjected to 120 mm Hg of pressure for 30 minutes. This pressure was selected based on normal physiological arterial pressure. During the incubation, vessels were perfused with oxygenated (95% O_2_, 5% CO_2_) KRB maintained at 37°C. Perfusion was accomplished using a peristaltic pump with the flow rate set at 11.1 ml/min resulting in a shear stress of ~0.5 dynes/cm^2^. The intraluminal pressure was controlled by adjusting the air pressure introduced into a fluid reservoir. The system was calibrated before all experiments. System pressure was monitored using pressure transducers (Gould model P231D) situated before entry and after exit from the mounted vessel. During the loading procedure, the pressure in the vessels was gradually raised in a step-wise fashion (10 mmHg every 1 min) to a mean arterial pressure of 120 mmHg.

### Immunoblot analysis

At the end of each experiment, the vessels were immediately snap-frozen in liquid nitrogen. To prepare protein isolates, venae cavae were pulverized under liquid nitrogen using a mortar and pestle and washed three times with ice cold phosphate buffered saline (PBS). Protein was extracted using T-PER (2 mL/1 g tissue weight) (Pierce, Rockford, IL) supplemented with 100 mM NaF, 1 mM Na_3_VO_4_, 2 mM PMSF 1 μg/ml aprotinin, 1 μg/ml leupeptin, and 1 μg/ml pepsatin as detailed by the manufacturer. After centrifugation (1000 g × 10 min), the supernatant was collected and the concentrations of homogenates were determined in triplicate via the Bradford method (Pierce) using bovine serum albumin as a standard. Samples were diluted to a concentration of 1.5 mg/mL in SDS-loading buffer and boiled for 5 minutes. Thirty μg of total protein for each sample were separated on a 10% or 15% SDS-PAGE gel, transferred onto Hybond nitrocellulose membranes (Amersham Biosciences, Piscataway, NJ) using standard conditions, and stained with Ponceau S to verify transfer and equal loading of lanes. Membranes were blocked in buffer (5% nonfat dry milk in TBST) for 1 hour at room temperature, washed (TBST, 3 × 5 min), and incubated in primary antibody overnight at 4°C. After washing to remove excess antibody (TBST, 3 × 5 min), membranes were incubated in horseradish peroxidase (HRP)-linked secondary antibodies for 1 hour at room temperature and rewashed (TBST, 3 × 5 min). Proteins were visualized by enhanced chemiluminescence (ECL) Western blotting detection reagent (Amersham Biosciences, Piscataway, NJ) and quantified by densitometry using a flatbed scanner (Epson Perfection 3200 PHOTO) and Imaging software (AlphaEaseFC). Exposure time was adjusted to keep the integrated optical densities within a linear and non-saturated range. Molecular weight markers (Cell Signaling) were used as mass standards and NIH 3T3 cell lysates were included as positive controls. Membranes were stripped with Restore western blot stripping buffer as detailed by the manufacturer. After verifying the absence of residual antibody binding by interrogating the membrane with the ECL reagent, membranes were washed and used for reprobing. To minimize potential experimental error associated with membrane stripping, the order of antibody incubations was randomized between experiments.

### Data analysis

Results are presented as mean ± SEM. Data were analyzed using the SigmaStat 3.0. A one-way analysis of variance (ANOVA) was performed for overall comparisons with the Student-Newman-Keuls post hoc test used to determine differences between groups. The level of significance accepted *a priori *was ≤ 0.05.

## Results

### Verification of loading stimulus

No apparent differences in gross morphology were noted at the light microscope level between venae cavae obtained from lean and obese Zucker rats (data not shown). Isolated vessels responded to stretch in a passive manner (data not shown). Vena cava loading pressure was constantly recorded throughout the loading procedure. If fluctuations in stretch induced loading occurred, the vessel was immediately discarded.

### Effect of diabetes on basal MAPK expression and phosphorylation

**Figure 1:**
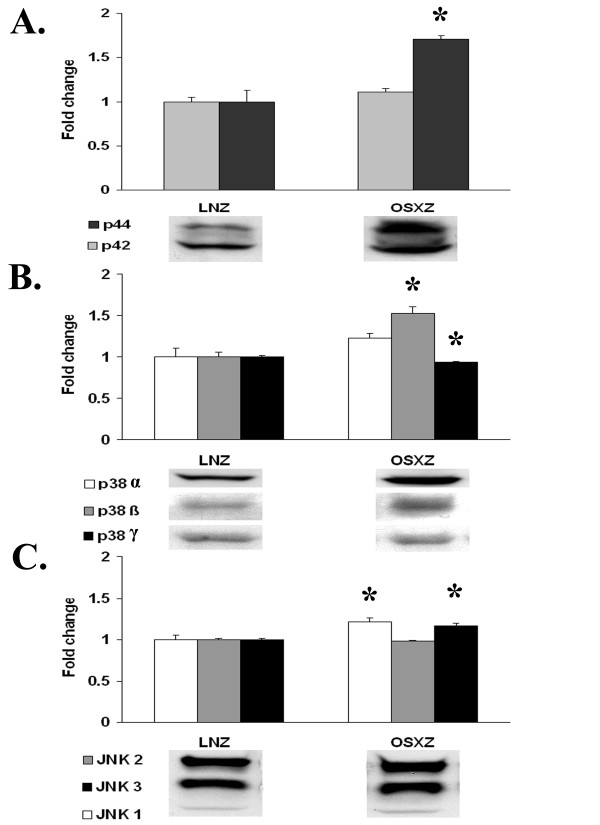
**MAPK expression in normal and diabetic rat inferior vena cava**. Immunoblot analyses indicating protein content of A) p44/42-MAPK, B) p38-MAPK, and C) JNK-MAPK in venae cavae from non-diabetic lean Zucker (LNZ) and diabetic obese syndrome X Zucker (OSXZ) rats. * Significantly different from the non-diabetic venae cavae, (P < 0.05). n = 6/group.

Previous reports have suggested that the MAPK proteins and in particular, p38 MAPK is associated with regulating load-induced caspase-3 activation in the venae cavae [7]. To investigate the influence of diabetes on the expression and regulation of MAPK proteins in the inferior vena cava, we used immunoblotting to determine the total amount of Erk1/2 (p44/p42), Jnk and p38-MAPK present in venae cavae obtained from normal and diabetic animals. We observed a 70.6 ± 16.4% higher basal expression of the Erk1 (p44)-MAPK in the inferior vena cava of diabetic animals compared that from the non-diabetic vena cava (P < 0.05), while no significant difference was detected in the basal expression of ERK 2 (p42) MAPK (Fig. 1A). In the diabetic IVC, the amount of p38β MAPK was 52.3 ± 11.8% higher (P < 0.05), the amount of p38γ MAPK was 6.1 ± 2.6% lower (P < 0.05); while p38α levels were not different (Fig. 1B). In a similar fashion, JNK 1- and JNK3-MAPK expression in the diabetic vena cava was 21.5 ± 9.3% and 16.8 ± 3.3% higher (P < 0.05) compared to the non-diabetic controls, while JNK2-MAPK levels were not altered (Fig. 1C).

**Figure 2:**
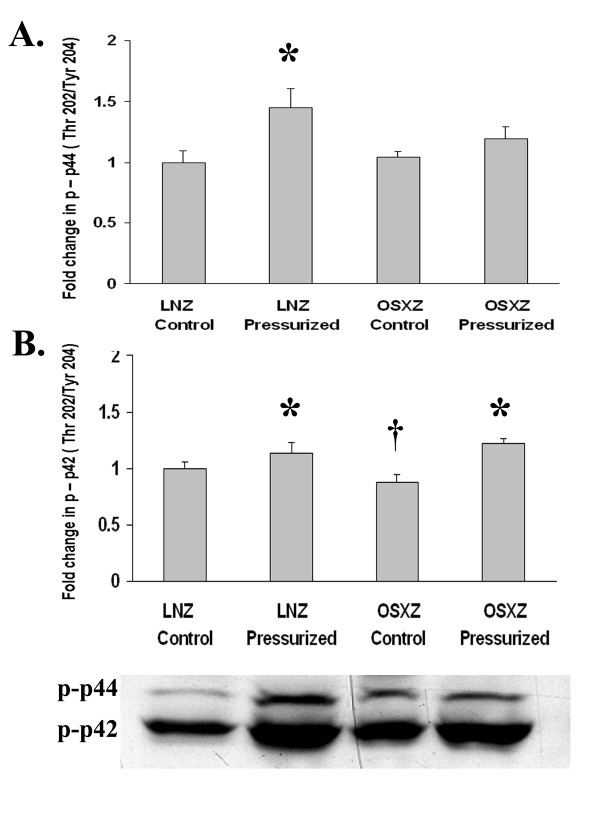
**Diabetes alters loading-induced p44/42 phosphorylation in the IVC**. The basal (control) and pressure-induced phosphorylation of the ERK1/2-MAPK in venae cavae from non-diabetic lean Zucker (LNZ) and diabetic obese syndrome X Zucker (OSXZ) rats. Phosphorylation status was calculated as phosphospecific optical density divided by the unpressurized non-diabetic control value. * Significantly different from unloaded venae cavae within the same group (P < 0.05). † Significantly different from corresponding LNZ venae cavae (P < 0.05). n = 6/group.

**Figure 3:**
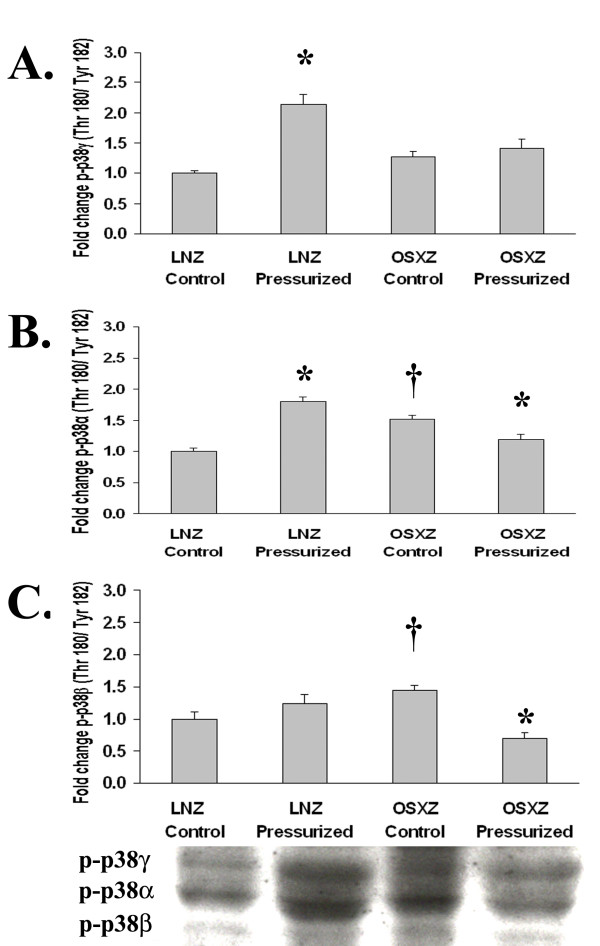
**Diabetes related alterations in loading-induced p38-MAPK phosphorylation in the IVC are isoform-specific**. The basal (control) and pressure-induced phosphorylation of the p38-MAPK in venae cavae from non-diabetic lean Zucker (LNZ) and diabetic obese syndrome X Zucker (OSXZ) rats. Phosphorylation status of A.) p38γ-MAPK, B.) p38α-MAPK, and C.) p38β-MAPK was calculated as phosphospecific optical density divided by the unpressurized non-diabetic control value. * Significantly different from unloaded venae cavae within the same group (P < 0.05). † Significantly different from corresponding non-diabetic venae cavae (P < 0.05). n = 6/group.

**Figure 4:**
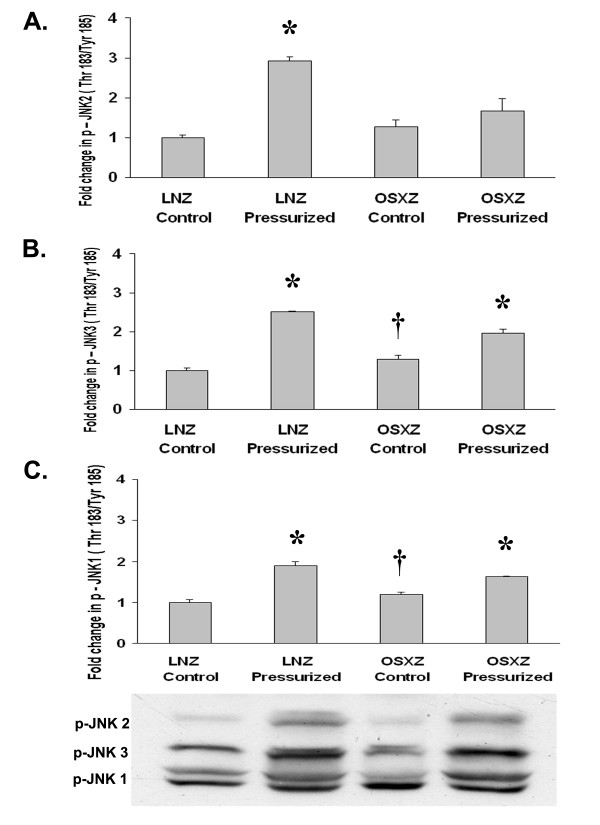
**Diabetes alters loading-induced Jnk phosphorylation in the IVC**. The basal (control) and pressure-induced phosphorylation of the JNK-MAPK in venae cavae from non-diabetic lean Zucker (LNZ) and diabetic obese syndrome X Zucker (OSXZ) rats. Phosphorylation status of A.) JNK2-MAPK, B.) JNK3-MAPK, and C.) JNK1-MAPK was calculated as phosphospecific optical density divided by the unpressurized non-diabetic control value. * Significantly different from unloaded venae cavae within the same group (P < 0.05). † Significantly different from corresponding non-diabetic venae cavae (P < 0.05). n = 6/group.

We next examined the influence of diabetes on the phosphorylation status of the MAPK family proteins in the inferior vena cava. The expression of phospho-ERK 1 (p44) (Thr202/Try 204) (Fig. 2A), phospho-p38γ (Thr180/Tyr182) (Fig. 3A), and phospho-JNK-2 (Thr183/Tyr185) (Fig. 4A) showed no change with diabetes. However, basal phospho-ERK 2 (p42) (Thr202/Tyr204) MAPK expression was lower in the diabetic inferior vena cava by 12.0 ± 11.46% (P < 0.05) (Fig. 2B). Expression of phospho-p38α (Thr180/Tyr182), phospho-p38β (Thr180/Tyr182), phospho-JNK1 (Thr183/Tyr185) and phospho-JNK3 (Thr183/Tyr185) was 52.3 ± 11.8%, 45.8 ± 18.2%, 19.4 ± 11.6% and 29.5 ± 17.6% higher in the diabetic IVC, respectively as compared to control (P < 0.05) (Fig. 3B, 3C, 4C and 4B).

### Effect of diabetes on pressure induced MAPK phosphorylation

We examined the effect of pressurization of inferior vena cava on ERK 1/2-, p38- and JNK-MAPK phosphorylation using immunoblot analyses. In inferior venae cavae subjected to 30 minutes of arterial pressure loading (120 mm Hg), phosphorylation of the ERK1 (p44) (Thr202/Tyr204)-MAPK increased 44.2 ± 25.2% in the non-diabetic rat venae cavae, while that in the diabetic rat venae cavae remained unchanged, (P < 0.05) (Fig. 2A). Expression of phospho-ERK 2 (p42) (Thr202/Tyr204)-MAPK increased 13.2 ± 5.3% and 39.0 ± 10.5% in the non-diabetic and diabetic rat inferior vena cava, respectively, (P < 0.05) (Fig. 2B). Pressure loading elicited an 80.8 ± 12.7% increase in the phosphorylation of p38α (Thr180/Tyr182)-MAPK in non-diabetic rat inferior vena cava. Conversely, p38α (Thr180/Tyr182)-MAPK phosphorylation in response to pressurization decreased by 21.3 ± 10.0% in the diabetic rat vena cava (P < 0.05) (Fig. 3B). Similarly, pressure induced phosphorylation of the p38β (Thr180/Tyr182)-MAPK demonstrated no significant change in the non-diabetic rat inferior vena cava, but was 52.0 ± 14.9% lower in the diabetic rat vena cava (P < 0.05) (Fig. 3C). Pressure induced phosphorylation of the p38γ (Thr180/Tyr182)-MAPK increased 114.1 ± 20.8% in the non-diabetic rat vena cava, while in the diabetic rat inferior vena cava it remained unchanged (P < 0.05) (Fig. 3A). Jnk1 (Thr183/Tyr185) phosphorylation increased in the non-diabetic and diabetic rat inferior vena cava by 89.9 ± 15.1% and 36.0 ± 5.6%, respectively, in response to pressure loading (P < 0.05) (Fig. 4C). Likewise, pressure induced phosphorylation of the Jnk2 (Thr183/Tyr185)-MAPK increased 192.6 ± 16.8% in the non-diabetic rat inferior vena cava while in the diabetic rat inferior vena cava JNK2 (Thr183/Tyr185) phosphorylation remained unchanged (P < 0.05) (Fig. 4A). JNK3 (Thr183/Tyr 85)-MAPK phosphorylation increased in the non-diabetic and diabetic rat inferior vena cava by 150.9 ± 9.5% and 50.4 ± 14.4%, respectively in response to pressure loading (P < 0.05) (Fig. 4B).

### Apoptotic signaling following venous loading is suppressed in diabetes

**Figure 5:**
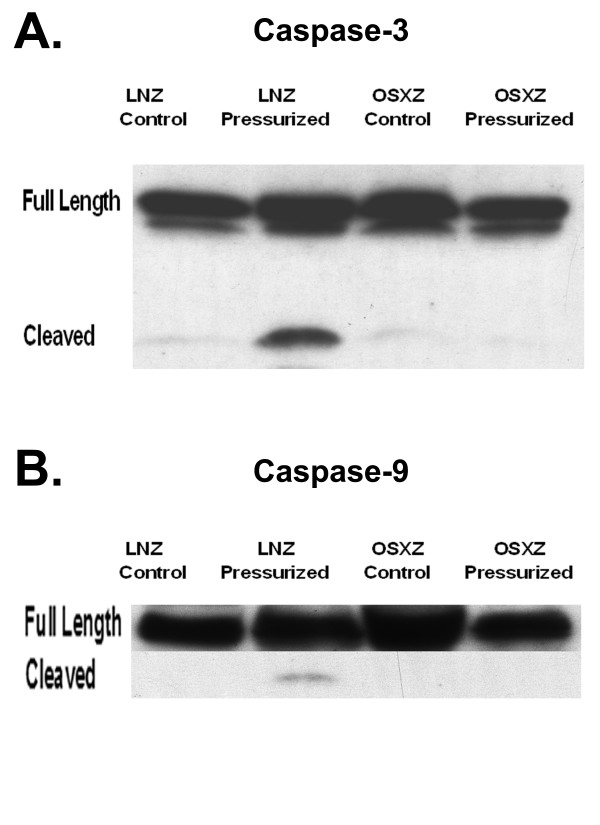
**The diabetic IVC does not exhibit loading-induced caspase activation**. The basal (control) and loading (pressure)-induced cleavage of A.) caspase-3 and B.) caspase-9 in venae cavae from non-diabetic lean Zucker (LNZ) and diabetic obese syndrome X Zucker (OSXZ) rats (n = 6/group).

Previous reports have suggested that the load-induced activation of caspase-3 is important in mediating apoptotic events following increased loading of the vena cava [7]. To examine possible differences in apoptosis-related signaling between non-diabetic and diabetic venae cavae, we measured differences in the regulation of caspase-3, caspase-9, bax and bcl-2 between these tissues. Caspase-3 is an endopeptidase that has been implicated in apoptosis. Because caspases are normally activated by proteolytic cleavage [8], we examined the effects of diabetes and increased loading on the amount of full length versus cleaved caspase-3 in vena cava samples obtained from non-diabetic and diabetic animals. Immunoblotting demonstrated no differences in the total amount of full-length capase-3 or caspase-9 between venae cavae of diabetic animals compared to that observed in the non-diabetic venae cavae (Figure 5A and 5B). The application of arterial pressure increased the amount of cleaved caspase-3 in the non-diabetic rat venae cavae to 276.0 ± 36.0%, whereas there was no change in the diabetic rats (P < 0.05) (Figure 5A). To confirm these findings, we investigated the regulation of caspase-9, the upstream activator of caspase-3. Immunoblot analysis showed a 85.8 ± 25.1% increase in cleaved caspase-9 upon pressurization of non-diabetic rat venae cavae (Figure 5B; P < 0.05). No caspase-9 cleavage was observed in the diabetic rat venae cavae upon pressurization.

**Figure 6:**
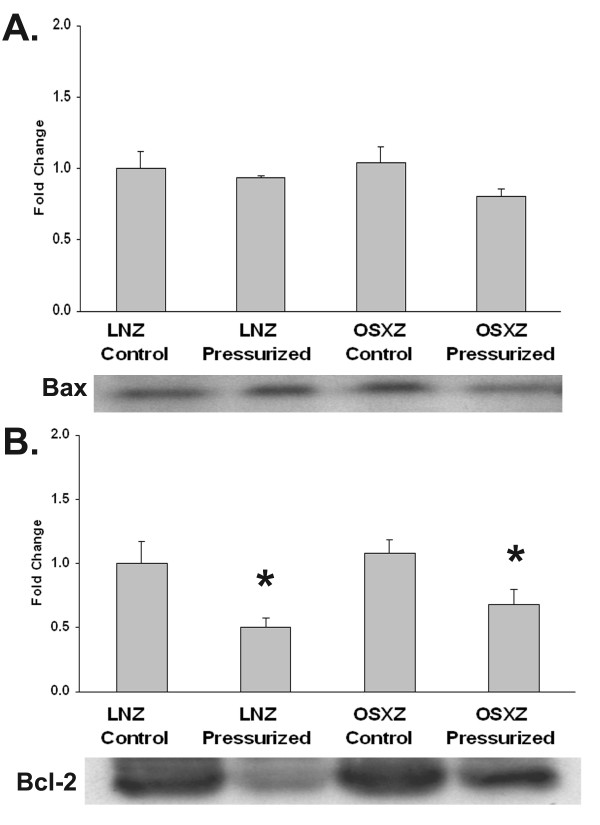
**Similar trends in Bax and Bcl-2 expression and loading-induced activity in normal and diabetic rat IVC**. Basal (control) and loading (pressure)-induced regulation of (A.) Bax and (B.) Bcl-2. in venae cavae from non-diabetic lean Zucker (LNZ) and diabetic obese syndrome X Zucker (OSXZ) rats. * Significantly different from unloaded venae cavae within the same group (p < 0.05). n = 6/group.

It is thought that the ratio of Bax to Bcl-2 plays an important role in regulating the release of cytochrome-c from the mitochondria into the cytosol with the release of cytochrome-c and cell death favored as the balance shifts toward Bax [9]. With diabetes, no difference in either the amount of Bax or Bcl-2 was noted (Figure 6). Unlike caspase-3 or -9, Bax levels were not altered with increased vena cava loading in either the non-diabetic or diabetic vena cava. Increased pressurization of the non-diabetic and diabetic venae cavae decreased Bcl-2 levels by 50.2 ± 24.2% and 55.9 ± 22.8%, respectively (P < 0.05) (Figure 6B).

### Akt regulation may be altered in the diabetic vena cava

**Figure 7:**
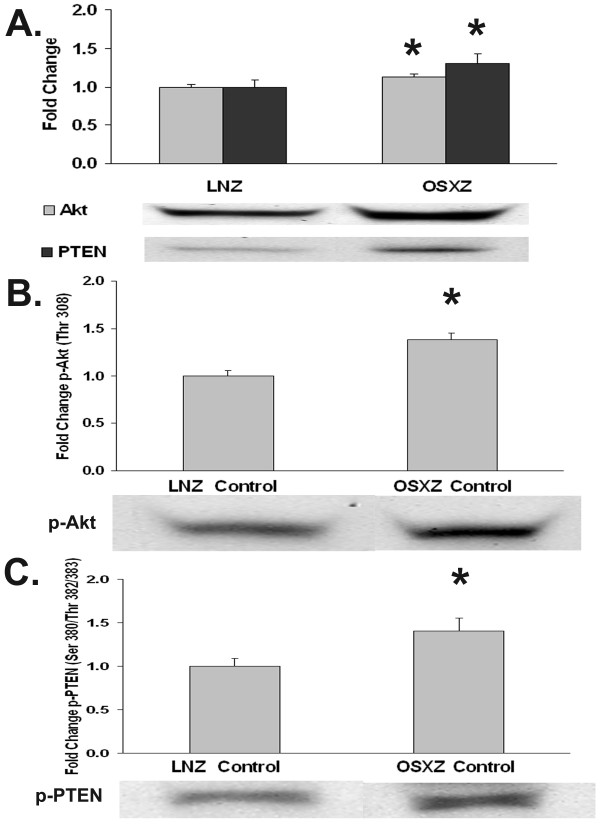
**Diabetic IVC shows higher expression and phosphorylation of Akt and PTEN**. Content of A) Akt and PTEN, B.) p-Akt, and C.) p-PTEN in vessels from non-diabetic lean Zucker (LNZ) and diabetic obese syndrome X Zucker (OSXZ) rats. Relative changes in protein levels were determined by immunoblotting analysis. * Significantly different from the non-diabetic venae cavae, (P < 0.05). n = 6/group.

**Figure 8:**
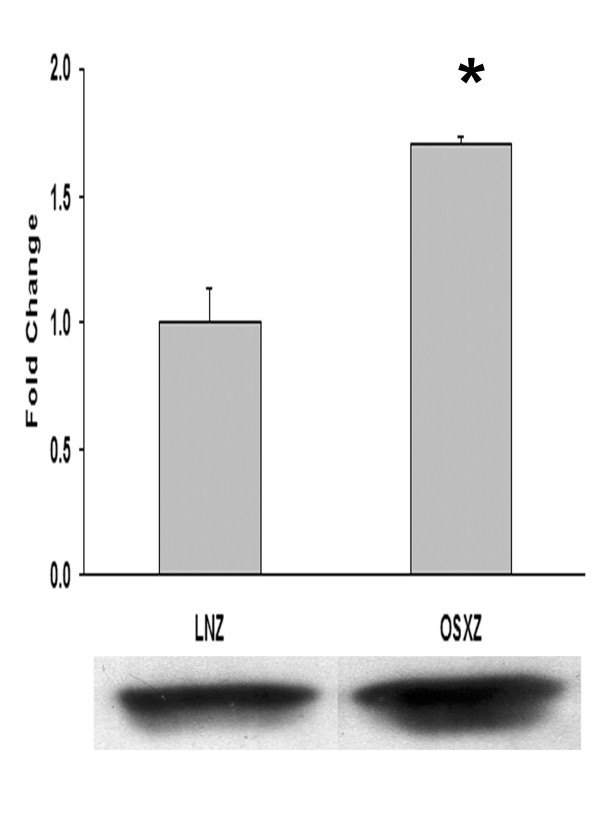
**Phospho-Bad (Ser136) expression is increased in the Diabetic IVC**. Vena cava content of p-Bad (Ser136) in non-diabetic lean Zucker (LNZ) and diabetic obese syndrome X Zucker (OSXZ) rats. * Significantly different from the non-diabetic venae cavae, (p < 0.05). n = 6/group.

In addition to the MAPKs the protein kinase B (Akt) pathway is thought to be an important regulator of cell apoptosis and replication [10,11]. To examine the influence of diabetes on the regulation of Akt, we compared the basal expression and phosphorylation of Akt in the venae cavae of non-diabetic and diabetic rats. Compared to non-diabetic animals, the IVC content of Akt was found to be 17.6 ± 9.9% and degree of basal Akt phosphorylation (Ser 308) was 38.3 ± 11.6% higher in the diabetic rats (P < 0.05) (Figure 7A). Because Akt phosphorylation has been shown to be regulated, in part, by PTEN, we examined whether diabetes influenced PTEN expression or its degree of basal phosphorylation. Compared to non-diabetic rats, the amount of PTEN and phosphorylated PTEN (Ser308/Thr382-383) in the diabetic venae cavae increased 31.4 ± 21.3 % and 41.0 ± 24.2 %, respectively (Figure 7A and 7C; P < 0.05). To confirm these apparent differences in Akt regulation with diabetes, we examined the regulation of Bad. Bad is a pro-apoptotic member of the Bcl-2 family and can bind to Bcl-xL in the unphosphorylated state promoting apoptosis. When phosphorylated at Ser136 by Akt, Bad is inactivated and can no longer promote apoptosis [12]. Immunoblotting analysis showed a 70.7 ± 15.1% higher basal expression of phospho-Bad (Ser136) in the venae cavae of diabetic rats as compared to non-diabetic rats (Figure 8). However, there was no difference in Bad phosphorylation between non-diabetic and diabetic venae cavae. Application of arterial pressure for 30 mins caused a 69.85 ± 26.4 % decrease in Bad phosphorylation in venae cavae of non-diabetic rats and a 75.2 ± 17.7% decrease in the venae cavae of diabetic rats (P < 0.05; data not shown).

## Discussion

Diabetes is known to be associated with cardiovascular disease and vein grafts in the diabetic population are often characterized by an increased vein graft neointimal hyperplasia along with significantly higher graft failure rates compared to non-diabetic patients [1]. Here, we report findings that suggest that the inferior venae cavae of normal and diabetic animals exhibit differences in the load-induced regulation of MAPK and apoptosis-related signaling. The data suggest that loading (stretch) of the non-diabetic vena cava is associated with the phosphorylation (activation) of ERK1 (p44)-, ERK2 (p42)-, p38α-, p38γ-, JNK1-, JNK2-, and JNK3-MAPK along with cleavage of capase-3 -9, and -12 (Figures 1, 2, 3, 4, 5). Conversely, in the diabetic vena cava, we failed to find any indication of caspase activation while the load-induced response of ERK1, JNK2, p38α-, p38β, and p38γ- was different from that seen in the non-diabetic vena cava (Figures 2, 3, 4, 5). Taken together, these results suggest that venous mechanotransduction processes may be altered with diabetes.

### Diabetes is associated alterations in vena cava MAPK regulation

In mammalian cells, the ERK1/2-, p38- and JNK-MAPK pathways are thought to be the three major signaling cascades comprising the MAPK signaling system [13]. The results of the present study indicate that diabetes is associated with changes in the how the venae cavae regulates MAPK pathways. When compared to the non-diabetic vena cava, the diabetic vena cava exhibited a higher content of p44-, p38β-, JNK1-, and JNK-3-MAPK and a diminished amount of p38γ-MAPK. To our knowledge these changes have not been demonstrated before. At the present time, neither the mechanism(s) responsible for these alterations nor the exact physiological significance of these changes is known.

Previous studies investigating the effect of increased glucose or the influence of diabetes on MAPK protein levels has been equivocal. Indeed, some studies have demonstrated no effect [14] while others have suggested that these conditions increase the expression of these proteins [15-17]. For example, Chen and colleagues recently demonstrated that streptozotocin-induced diabetes is associated with increases in the amount and phosphorylation (activation) of p38-MAPK [18]. Similarly, the exposure of cultured endothelial progenitor cells to high glucose has been shown to increase p38-MAPK phosphorylation in a dose-dependent manner [19]. Whether the alterations in p38-MAPK regulation observed in these studies was due to the differential regulation of different p38-MAPK isoforms is unclear as it appears that the antibodies used in these investigations did not appear to be able to differentiate between the different p38-MAPK family members. The data of the present are in agreement with these findings and further, suggest that both the expression and basal phosphorylation of p38β-MAPK is higher in the diabetic vena cava.

Previous reports have suggested that p38β-MAPK can oppose apoptosis [20-22] suggesting that some form of cellular pre-conditioning to resist apoptosis may occur in diabetic tissues. Why diabetes may be associated with differences in the regulation of some p38-MAPK isoforms and not others awaits further clarification. Similarly, we observed that the expression and basal phosphorylation of Jnk1 and Jnk3, but not Jnk2, were higher in the diabetic vena cava when compared to vessels obtained from non-diabetic animals. Obesity has been linked with increased endoplasmic reticulum stress, which leads to suppression of insulin receptor signaling via hyperactivation of Jnk and subsequent serine phosphorylation of IRS-1 [23]. Accordingly, it is possible that the higher basal levels of JNK may represent some aspects of this response in the obese diabetic Zucker rats. Further research is required to more fully address this hypothesis.

Similar to the MAPK signaling cascade, the regulation of Akt signaling also appears to be altered in the diabetic vena cava. Akt is thought to mediate the effects of PI 3-kinase on some cellular events, such as apoptosis and protein synthesis [24,25]. PTEN is a phosphatase that has been proposed to inhibit the PI3K/Akt and ERK 1/2-MAPK signaling cascades and function in the control of cell cycle arrest [26,27]. The ability of PTEN to inhibit the PI3K/Akt and ERK 1/2-MAPK signaling cascades is thought to be negatively regulated by phosphorylation [28]. Our results indicate that the vena cava content and degree of basal Akt and PTEN phosphorylation was increased in diabetic compared to non-diabetic rats (Figure 7). Previous reports have suggested that the amount of Akt protein has been found to decrease in the diabetic mice aorta [29], not change in diabetic rat heart [30,31] or exhibit a tendency to increase in diabetic skeletal muscle [32]. Our finding that the basal phosphorylation of PTEN is increased (Figures 7) in the vena cava of diabetic rats is consistent with our data demonstrating increased Akt basal phosphorylation and previous findings demonstrating that alterations in PTEN are associated with the development of insulin resistance [33,34]. Future experiments examining the content of these molecules in other tissues may help clarify whether the changes we observed are causative, compensatory or merely associated with diabetes.

### Pressure-induced activation of MAPK and apoptosis associated proteins is altered in the vena cava of obese Zucker rats

Using a similar *ex-vivo *vena cava preparation, Goldman and colleagues recently presented data suggesting that mechanical stretch may play an important role in regulating venous graft failure [7]. Further, these authors suggested that increased loading of isolated venae cavae from non-diabetic rats was characterized by the p38-MAPK dependent activation of caspase-3 which in turn, was associated with α-actin degradation. The findings of the present investigation support this contention. In addition, the present data suggest that several differences exist in the way that venae cavae from non-diabetic and diabetic animals "sense and respond" to mechanical loading. For example, unlike what was seen in the normal vena cava, increased loading in the diabetic vena cava was associated with an inability of this tissue to increase the phosphorylation of ERK1-, JNK-2, or any of the p38-MAPKs (Figures 2, 3, and 4). These differences in the ability to activate the p38α- and JNK-2-MAPK proteins may be of particular interest. Recent studies have suggested that these proteins may play key roles in mediating the induction of cell death and in the activation of caspases [35-39]. Our findings that the activation of caspase-3 and -9 appear to differ following a loading stimulus in the vena cava of diabetic vena cava (Figures 5) support this possibility.

In addition to the MAPK proteins, Akt has also been shown to directly affect pro-apoptotic molecules. Bad and Caspase-9 are thought to be key regulators of cellular apoptosis whose activity is regulated, in large part, by Akt phosphorylation [25,40,41]. Akt phosphorylates pro-caspase-9 at Ser196, which inhibits the proteolytic processing of this molecule. We show increased basal Akt expression and phosphorylation in the vena cava of diabetic rats as compared to non-diabetic rats and that this alteration appears to be associated with an inability of the diabetic vena cava to activate (cleave) caspase-9 and caspase-3 following the application of arterial pressure (Figure 5). Whether these events are directly linked will require further investigation.

Like the caspases, Bad is a pro-apoptotic member of the Bcl-2 family which is thought to bind Bcl-xL in the unphosphorylated state promoting apoptosis [42]. Akt has been shown to inhibit this process thereby promoting cell survival via its ability to phosphorylate Bad at Ser136 [12]. We see a markedly high basal phosphorylation of Bad (Ser136) in the diabetic vena cava as compared to that seen in non-diabetic vessels (Figure 8), which is suggestive of a heightened anti-apoptotic activity in the diabetic tissue. Whether this alteration in Bad phosphorylation is directly related to the differences in caspase-3 and -9 cleavage we see between the diabetic and non-diabetic vena cava awaits clarification.

## Conclusion

In summary, diabetes has been associated with increased prevalence of vein graft failure. Compared to what we observe in the non-diabetic vena cava, the regulation of the MAPK, Akt, and PTEN proteins appears differ in the diabetic vena cava possibly resulting in a net protective effect against pressure induced apoptosis-related signaling. Whether alterations in MAPK- or Akt-related signaling alone or other molecules contribute to the load-induced differences in apoptotic-related signaling we observe in the diabetic venae cavae remains to be determined. Given the relatively small number of animals employed in the present study (n = 12/group) caution regarding the extrapolation of the findings presented here to a human population is warranted. Nonetheless, the data of this study may be useful towards elucidating the cellular and molecular mechanisms that may underlie the increased problems associated with vein grafts in the diabetic population and underscores the basis for future studies investigating the role that MAPK and Akt may play in vein failure.

## Authors' contributions

K.M. R. and D. H.D. contributed equally to this study, performed the vessel loading experiments, participated in the experimental design, immunoblotting experiments, and performed the data analysis and manuscript preparation; S.K., A.K., and D. L. P. participated in the loading experiments and performed the immunoblotting experiments; P. W. participated in the data analysis and manuscript preparation; E. R. B. participated in all experimental aspects and manuscript preparation. All authors have read and approved the final manuscript.
